# CORRECTION

**DOI:** 10.1111/cas.15898

**Published:** 2023-07-03

In an article^1^ titled “Long non‐coding RNA NEAT1 promoted ovarian cancer cells' metastasis through regulation of miR‐382‐3p/ROCK1 axials” by Yangcheng Liu, Yong Wang, Xinming Fu, and Zhi Lu, there were errors in 2D and 2E:

The corrected Figure 2 is shown here:

**FIGURE 2:**
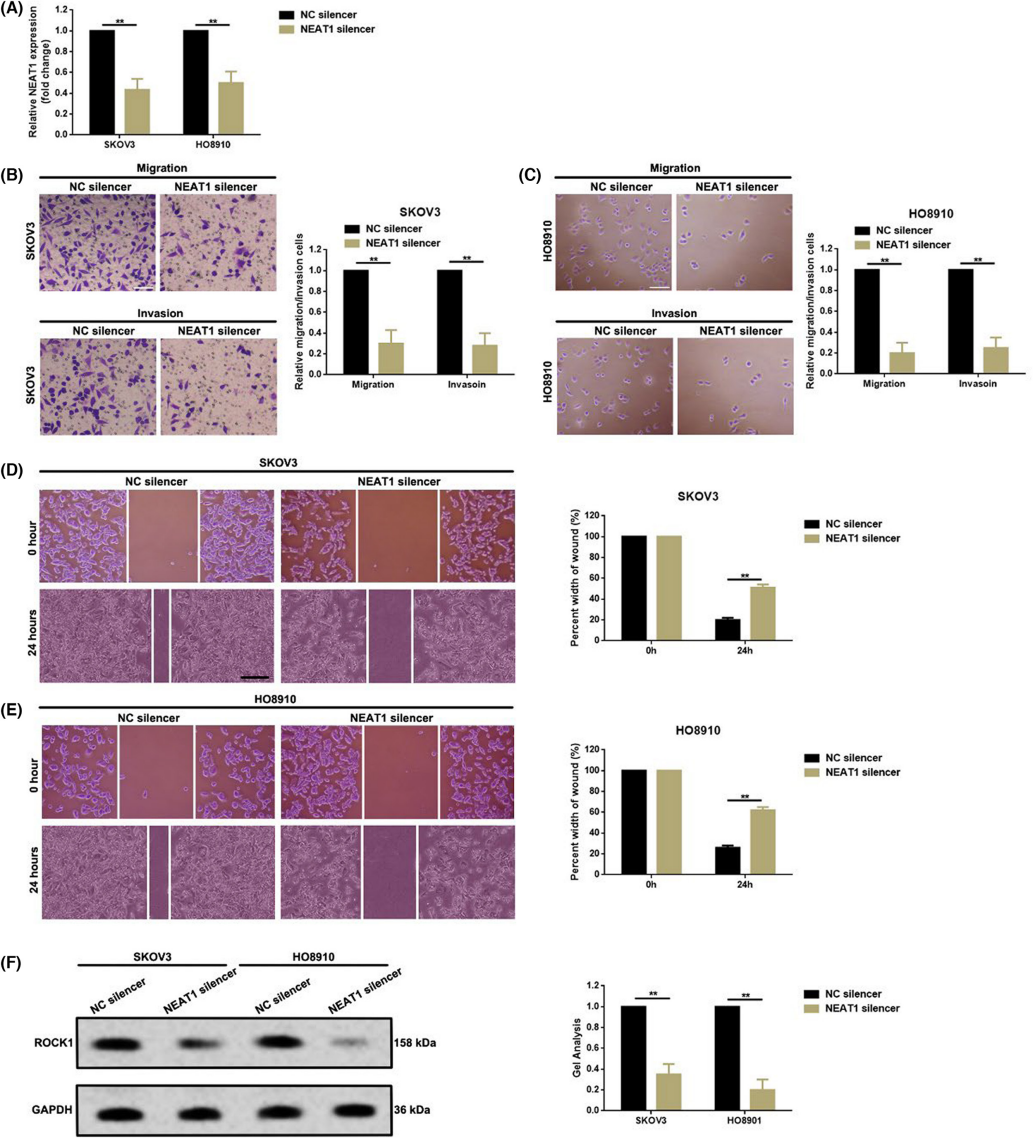


The authors apologize for the errors.
